# Age Differences in Factors Associated With Insomnia Severity: A Comparative Study of Questionnaire Scores and Heart Rate Variability Parameters

**DOI:** 10.1155/da/7711037

**Published:** 2026-07-03

**Authors:** Kong Xinyu, Guo Xiaodan, Wang Mengmeng, Zhang Haodong, Xiao Fulong

**Affiliations:** ^1^ Department of Sleep Medicine, Peking University People’s Hospital, Beijing, China, pku.edu.cn; ^2^ Nursing Department, Sir Run Run Shaw Hospital, School of Medicine, Zhejiang University, Hangzhou, Zhejiang, China, zju.edu.cn

**Keywords:** age differences, autonomic nervous system, heart rate variability, primary insomnia, questionnaire scores

## Abstract

**Objective:**

Primary insomnia exhibits diverse clinical profiles across age groups, yet the underlying mechanisms remain unclear. This study aimed to explore age‐related differences in factors associated with insomnia severity.

**Methods:**

Evaluating questionnaire scores and heart rate variability (HRV) parameters in young adult age (18–44 years, *n* = 124) and old adult age (≥45 years, *n* = 120) patients with primary insomnia. All participants underwent comprehensive assessments using the Athens Insomnia Scale (AIS), Insomnia Severity Index (ISI), and multiple psychological scales, alongside HRV analysis to evaluate autonomic nervous system function.

**Results:**

Significant between‐group differences were observed in Morningness–Eveningness Questionnaire (MEQ) scores and State–Trait Anxiety Inventory (STAI) state anxiety scores, with young adult patients showing a higher prevalence of evening chronotype. All HRV parameters except high‐frequency (HF) power differed significantly between age groups, indicating age‐related autonomic dysregulation. Regression analyses identified distinct predictors of insomnia severity: insomnia severity in young adults was independently associated with depressive symptoms and chronotype, whereas that in older adults was predicted by anxiety symptoms and HRV parameters reflecting autonomic imbalance (low‐frequency [LF]/HF ratio, mean R–R intervals [RRIs]).

**Conclusion:**

These findings highlight age‐dependent associations between insomnia severity, psychological factors, and autonomic function, suggesting that young adult insomnia may be more linked to depressive symptoms and chronotype, whereas old adult insomnia is strongly associated with anxiety and autonomic imbalance. This may inform age‐tailored therapeutic strategies for primary insomnia.

**Summary**

• Primary insomnia severity may be associated with age, with younger groups linked to depressive symptoms and chronotype and older groups tied to anxiety and autonomic imbalance.
• Heart rate variability (HRV) parameters, particularly the low‐frequency (LF)/high‐frequency (HF) ratio, differ significantly between age groups, suggesting age‐related autonomic nervous system dysregulation in insomnia.
• Evening chronotype prevalence is higher in young adult‐age insomnia patients, highlighting circadian rhythm differences as a potential contributor to age‐specific insomnia phenotypes.

## 1. Introduction

Insomnia disorder, characterized by difficulty initiating sleep, maintaining sleep, or early‐morning awakenings, alongside significant daytime impairment, is a prevalent sleep disorder affecting ~10%–30% of the general population [1]. Chronic insomnia not only significantly impairs quality of life, cognitive function, and daily performance but also increases the risk of developing psychiatric disorders, such as anxiety and depression, and physical conditions, including cardiovascular diseases [1]. Given its far‐reaching consequences, understanding the underlying factors contributing to insomnia severity is crucial for developing effective therapeutic strategies.

Previous research has suggested that age may play a pivotal role in the manifestation and mechanisms of insomnia [2–4]. Insomnia prevalence varies by age, with estimates of 15%–25% in young adults [5] and 20%–40% in older adults [6], reflecting age‐related physiological and psychosocial changes that may alter insomnia phenotypes. Age‐related physiological changes, such as alterations in circadian rhythms [7] and autonomic nervous system function [8], can impact sleep regulation. For instance, older adults often exhibit a natural shift toward morningness and reduced sleep efficiency, which may be associated with changes in the suprachiasmatic nucleus, the master circadian pacemaker [9]. Additionally, the autonomic nervous system, which controls physiological arousal during sleep, shows age‐dependent modifications; older individuals tend to have reduced parasympathetic activity and an altered sympathetic‐vagal balance, potentially contributing to sleep disturbances [10]. However, the specific factors associated with insomnia severity in different age groups remain incompletely understood, and few studies have directly compared younger and older adults with primary insomnia.

Heart rate variability (HRV), an established biomarker of autonomic nervous system function, has been increasingly used to investigate the physiological underpinnings of sleep disorders [11]. Higher HRV typically reflects better autonomic flexibility and physiological resilience, while reduced HRV has been linked to elevated stress levels and sleep fragmentation [11]. In insomnia patients, abnormal HRV patterns have been reported, indicating dysregulation of the autonomic nervous system [11]. Nevertheless, whether these HRV alterations vary by age and how they contribute to insomnia severity in different age cohorts remain unclear.

Furthermore, psychological factors, including anxiety and depression, are strongly associated with insomnia [1]. Younger adults may experience more situational stressors related to career development, social relationships, and family responsibilities, potentially exacerbating anxiety and sleep problems [12]. In contrast, older adults might face cumulative lifetime stress, age‐related health concerns, and social isolation, which could contribute to chronic anxiety and depressive symptoms—key correlates of insomnia [13]. However, the relative impact of psychological factors on insomnia severity across different age groups has not been comprehensively explored.

To address these knowledge gaps, the present study aimed to compare the clinical characteristics, psychological profiles, and HRV parameters between younger and older adults with primary insomnia. We focused on primary insomnia to isolate age‐related mechanisms specific to sleep regulation, rather than those secondary to comorbid conditions (e.g., chronic pain and depression) or other sleep disorders (e.g., obstructive sleep apnea syndrome). Secondary insomnia often reflects overlapping pathophysiology with underlying diseases, which could confound the associations between age, psychological factors, HRV, and insomnia severity. By excluding secondary insomnia, we aimed to identify pure age‐dependent patterns that may inform targeted treatments for primary insomnia. We hypothesized that age groups would differ in factors associated with insomnia severity, with psychological factors and circadian rhythm‐related variables being more prominent in younger patients and autonomic nervous system function and chronic psychological distress playing a more significant role in older patients. By using multiple validated questionnaires and HRV analysis, this study sought to identify age‐specific predictors of insomnia severity, providing insights into targeted therapeutic approaches for different age cohorts.

## 2. Materials and Methods

### 2.1. Patients

This was a cross‐sectional observational study conducted between October 2024 and June 2025 in the Department of Sleep Medicine, Peking University People’s Hospital. A total of 244 patients with primary insomnia were enrolled in the study (70 men and 174 women; mean age 46.9 ± 15.1 years; range 18–79 years). Informed consent was received from all subjects prior to the commencement of the study, and the protocol was approved by the Institutional Review Board of Peking University People’s Hospital.

Participant interview procedures: all participants completed a 30‐min structured interview conducted by a trained sleep researcher. The interview included verification of medical/psychiatric history, medication use, and sleep‐related symptoms, as well as confirmation of the primary insomnia diagnosis by a board‐certified sleep physician (Xiao Fulong) based on the International Classification of Sleep Disorders, 3rd Edition.

The inclusion criteria for the primary insomnia were the following: aged 18–80 years; complaints of difficulty falling and/or staying asleep for at least 3 months; persistence of symptoms despite adequate sleep opportunities; and daytime dysfunction (including mood disturbance, cognitive impairment, social or occupational difficulties). Criteria for exclusion were the following: a history of mental illness, depression or anxiety episodes, or presence of suicidal ideation; comorbid other sleep disorders (e.g., sleep apnea syndrome, restless‐legs syndrome, or narcolepsy); use of medications known to affect sleep within 2 weeks prior to testing (e.g., antidepressants and antipsychotics); excessive alcohol or caffeine intake.

Participants taking cardiovascular drugs (e.g., beta‐blockers), antidepressants, pain medications, hypnotics, or other psychoactive drugs were excluded because these agents directly modulate autonomic nervous system function (e.g., beta‐blockers reduce HRV), sleep architecture (e.g., hypnotics alter sleep stages), or psychological states (e.g., antidepressants affect anxiety/depression scores). Melatonin was also excluded as it regulates circadian rhythms. While this exclusion limits generalizability to medication‐using populations, it was necessary to isolate the direct associations between age, intrinsic psychological factors, HRV, and insomnia severity.

#### 2.1.1. Rationale for Inclusion and Exclusion Criteria

The inclusion and exclusion criteria were designed to prioritize internal validity—minimizing confounding variables that could obscure the direct associations between age, HRV, psychological factors, and insomnia severity (the core research objective). The primary goal of this study was to identify age‐specific mechanisms linking HRV, psychological factors, and insomnia severity in primary insomnia. Given the complexity of confounding variables (comorbidities, medications, and other sleep disorders) that co‐occur with insomnia, especially in older adults, prioritizing internal validity was necessary to avoid false‐positive or obscured associations between age and the outcomes of interest. While this design limits the generalizability of our findings to “ideal” primary insomnia patients (without comorbidities or medication use), it provides a critical foundation for understanding the intrinsic age‐related pathophysiology of primary insomnia.

### 2.2. Questionnaires Evaluation

#### 2.2.1. Athens Insomnia Scale (AIS)

The AIS was used to assess the severity of insomnia using diagnostic criteria determined by the International Classification of Diseases‐10 (ICD‐10) [14]. The questionnaire consists of eight questions that assess the onset of sleep, nighttime and early‐morning awakenings, sleep time, sleep quality, the frequency and duration of complaints, and complaints caused by insomnia and disturbances in daily functioning. Cronbach’s *α* = 0.81, test–retest reliability *r* = 0.8, and concurrent validity with polysomnography (PSG) were confirmed [1].

#### 2.2.2. Insomnia Severity Index (ISI)

The ISI is a widely used seven‐item questionnaire that focuses on the Diagnostic and Statistical Manual of Mental Disorders (DSM), 4th edition diagnostic criteria for insomnia, particularly created to assess how patients perceive insomnia and how it affects their quality of life, ability to operate daily and to maintain their sleep, and their level of concern about their sleeping problems [15]. Cronbach’s *α* = 0.83, test–retest reliability *r* = 0.79, and construct validity were supported by correlations with sleep quality [1].

#### 2.2.3. Epworth Sleepiness Scale (ESS)

The ESS is a subjective, self‐reported measurement of sleepiness. It measures the propensity to sleep under certain environmental conditions in daily life in recent weeks [16]. There are eight items corresponding to eight conditions. These items are rated on a scale of 0–3, with a score of N10 indicating excessive daytime sleepiness (EDS). Cronbach’s *α* = 0.81, test–retest reliability *r* = 0.74, and concurrent validity with PSG were confirmed [17].

#### 2.2.4. Hamilton Anxiety Rating Scale (HAMA)

The HAMA was used to measure the severity of anxiety symptoms. The participants were asked to rate 14 items on a Likert scale (0–4), where <17 indicates mild anxiety, 18–24 indicates mild to moderate anxiety, 25–30 indicates moderate to severe anxiety, and >30 indicates severe anxiety [18]. Cronbach’s *α* = 0.93; interrater reliability *r* = 0.91; and criterion validity with clinical diagnosis [19].

#### 2.2.5. Hamilton Depression Rating Scale (HAMD)

The HAMD is designed to measure the severity of depression symptoms [20]. It is administered by an interviewer, and in this study, a 17‐item version of the questionnaire was utilized. Scores ranging from 0 to 7 indicate no depression, scores between 8 and 15 suggest mild depression, scores between 16 and 28 indicate moderate depression, and scores of 29 and above indicate severe depression. Cronbach’s *α* = 0.88, interrater reliability *r* = 0.84, and criterion validity with clinical diagnosis [19].

#### 2.2.6. Fatigue Scale‐14 (FS‐14)

The FS‐14 was used to assess fatigue [21]. The scale has 2 dimensions and 14 items, including physical fatigue and psychological fatigue. The points for items number 1–8 are added to the physical fatigue scores, the points for items number 9–14 are added to the psychological fatigue scores, and the scores for all items were added to the total fatigue scores. The scale scores range from 0 to 14 points, and the higher the scores are, the worse the fatigue. Cronbach’s *α* = 0.80, test–retest reliability *r* = 0.79, and discriminant validity were between fatigued and nonfatigued groups.

#### 2.2.7. Morningness–Eveningness Questionnaire (MEQ)

The MEQ is one of the most used in chronobiological and chronopsychological research [22]. It consists of 19 mixed format items designated to assess an individual’s preferred timing of daily activities, particularly sleep and wake behaviors. The questionnaire included both Likert‐type and time‐based questions. Likert‐type items offer four options, with lower scores indicating a stronger evening preference. Time‐based items were scored based on selected time intervals over a 7‐h range, with all responses scored from 1 to 5. The total score is the sum of all item scores, and it is used to classify chronotype into five categories: definitely morning type (70–86), moderately morning type (59–69), neither type (42–58), moderately evening type (31–41), and definitely evening type (16–30). Cronbach’s *α* = 0.89, test–retest reliability *r* = 0.88, and structural validity were confirmed via factor analysis [23].

#### 2.2.8. State–Trait Anxiety Inventory (STAI)

The STAI is a self‐administered, Likert‐type instrument. It contains two separate 20‐item scales that determine anxiety in a specific situation (transient and situationally conditioned) and as a trait character (understood as a relatively permanent personality trait) [24]. The state scale of the STAI evaluates how respondents feel at a particular moment, and the trait scale refers to the habitual tendency to be anxious over a long period of time. Cronbach’s *α* = 0.84; test–retest reliability *r* = 0.81; and construct validity supported by correlations with psychological distress [25].

#### 2.2.9. Trait Coping Style Questionnaire (TCSQ)

Coping styles were measured using the TCSQ [26]. It is divided into two dimensions: positive coping style (PC) and negative coping style (NC), each consisting of 10 items, totaling 20 items. Responses range from “definitely not” to “definitely yes” with scores of 1–5 points, respectively. The scores for each dimension reflect the positive and negative attitudes and behavioral characteristics of the subjects when facing challenging setbacks. Cronbach’s *α* = 0.69 (negative) and *α* = 0.7 (positive); test–retest reliability *r* = 0.75 (negative) and *r* = 0.73 (positive); construct validity supported by correlations with psychological distress [27].

Moreover, all patients were evaluated for the symptoms and clinical signs of OSAS using the Berlin Questionnaire [28]. The Berlin Questionnaire classifies patients according to the risk of sleep apnea into two groups: low‐risk or high‐risk. Clinical evaluation for symptoms of restless leg syndrome using established diagnostic criteria based on the International Classification of Sleep Disorders, 3rd Edition.

### 2.3. HRV Data Collection and Analysis

HRV, respiration, and skin conductance were measured at rest seated during a 6 min period, using an EKG‐based analyzer (Intelligent Feedback Training System 2.0, JingShiBoRen, Beijing, China), with a sampling rate of 1000 Hz. Prior to recording, participants rested in a seated position for 10 min to stabilize autonomic function, and recordings were conducted in a quiet, temperature‐controlled room (22–24°C) to minimize artifacts. Participants were instructed to breathe normally (12–16 breaths/min) and avoid talking or movement during recording. Although we used a 6‐min recording duration (consistent with previous reports [29]), we acknowledge that 10‐min recordings are preferred to ensure ≥5 min of artifact‐free data. All recordings were visually inspected by a trained researcher to exclude segments with motion artifacts, dropped beats, or irregular breathing.

HRV analysis was performed both in a time domain and in a frequency domain. The following parameters were considered in the time domain: mean of R–R intervals (RRIs), the standard deviation of RRIs (SDNN), the root mean square of RRIs (SDSD), the root mean square of successive RRI differences (RMSSD), number of consecutive RRIs differing by >50 ms (NN50), and the percentage value of NN50 intervals (pNN50). SDNN reflects total HRV, whereas pNN50 and RMSSD reflect parasympathetic activity [30]. The frequency domain HRV measures include high‐frequency (HF power: 0.15–0.4 Hz), low‐frequency (LF power: 0.04–0.15 Hz), and very‐LF (VLF power: 0.003–0.04 Hz), as well as total power (TP), which were obtained in absolute power. With the use of appropriate autonomic blocking agents and experimental supine‐standing strategies, LF power is thought to be modulated by both sympathetic and parasympathetic activities, whereas HF power is mainly modulated by parasympathetic activity. The LF/HF ratio was computed as a measure of the sympathetic‐vagal balance toward sympathetic activity [31].

### 2.4. Statistical Analysis

According to previously published studies and clinical physiological characteristics, all patients were classified into two groups based on age: the young adult group (18–44 years) and the old adult group (45+ years) [32, 33]. Age 45 years was selected as the cutoff because it represents a critical threshold for significant age‐related changes in circadian rhythms, autonomic nervous system function, and sleep regulation, which are the key variables of this study. This grouping also ensured balanced sample sizes between the two cohorts for robust statistical comparisons. Prior to between‐group comparisons, the Shapiro–Wilk test was used to examine the normality of continuous variables, and Levene’s test was used to evaluate homogeneity of variance. For continuous variables with normal distribution and homogeneity of variance, Student’s *t*‐test (parametric test) was applied; for variables violating normality or variance heterogeneity, the Mann–Whitney *U* test (nonparametric test) was used. Categorical variables (e.g., chronotype and insomnia severity classification) were compared using the Chi‐squared test. Normally distributed continuous variables were presented as mean ± standard deviation (mean ± SD), nonnormally distributed continuous variables as median (interquartile range [IQR]), and categorical variables as count (percentage).

Prior to the analysis, all variables were screened for missing values. The missing rate of all data was less than 5%, and complete‐case analysis (listwise deletion) was used to handle missing data as this method is reliable for datasets with minimal missingness.

To identify factors associated with insomnia, we employed a two‐step statistical approach in two groups, respectively. The dependent variable was the AIS or ISI score, two gold‐standard scales for assessing insomnia severity with high reliability and validity, which directly reflect the core outcome of this study. While the independent variables were selected based on clinical evidence and pathophysiological relevance to primary insomnia, including (1) demographic information (e.g., age and gender); (2) scores from other questionnaires (e.g., ESS, HAMA, and HAMD); and (3) HRV parameters (e.g., SDNN, RMSSD, HF, and LF). Given high dimensionality and potential collinearity among independent variables, least absolute shrinkage and selection operator (LASSO) regression was used to screen clinically and statistically meaningful predictors before forward stepwise linear regression. This method was chosen to handle potential high‐dimensionality and collinearity among independent variables, leveraging a regularization penalty to shrink less relevant variables’ coefficients to zero, thereby selecting a parsimonious set of predictors. Independent variables with a regression coefficient equal to zero after the shrinkage process are excluded from the model, while independent variables with a nonzero regression coefficient are most strongly associated with the dependent variable. Based on the type measure of ‐2log‐likelihood and binomial family, the LASSO regression analysis running in R software runs 10 times K cross‐validation for centralization and normalization of included variables and then picks the best lambda value. “Lambda.lse” gives a model with good performance but the least number of independent variables. In the second step, using the subset of variables selected by LASSO, we then performed forward stepwise linear regression in SPSS to build a final model. This iterative procedure systematically added variables to identify the optimal set of predictors significantly associated with AIS or ISI score in two groups, respectively. The result was a linear regression model explicitly specifying which independent variables were independently related to the score of AIS or ISI, accounting for potential confounding effects and ensuring statistical robustness. LASSO regression was performed using R software (Version 4.2.1) with the glmnet package. Descriptive statistics, between‐group comparisons, and forward stepwise linear regression were analyzed using IBM SPSS Statistics (Version 26.0).

## 3. Results

### 3.1. Baseline Characteristics

Among 244 insomnia patients, 124 subjects were classified as the young adult group (38 males [30.6%], 86 females [69.4%], mean age 34.16 ± 6.92 years), and the other 120 subjects were classified as the old adult group (36 males [30%], 84 females [70%], mean age 60.24 ± 8.52 years). Among 244 insomnia patients, 107 patients were classified as having moderate insomnia, and 51 patients were classified as having severe insomnia based on the AIS score. While 117 patients were classified as having moderate insomnia and 48 patients were classified as having severe insomnia based on the ISI score. In young adult patients, 59 patients were classified as having moderate insomnia, and 19 patients were classified as having severe insomnia based on the AIS score. While 60 patients were classified as having moderate insomnia and 24 patients were classified as having severe insomnia based on the ISI score. In old adult patients, 48 patients were classified as having moderate insomnia, and 32 patients were classified as having severe insomnia based on the AIS score. While 57 patients were classified as having moderate insomnia and 24 patients were classified as having severe insomnia based on the ISI score.

### 3.2. Differences Between Two Groups of Insomnia Patients in Questionnaire Scores and HRV Parameters

**Figure 1:**
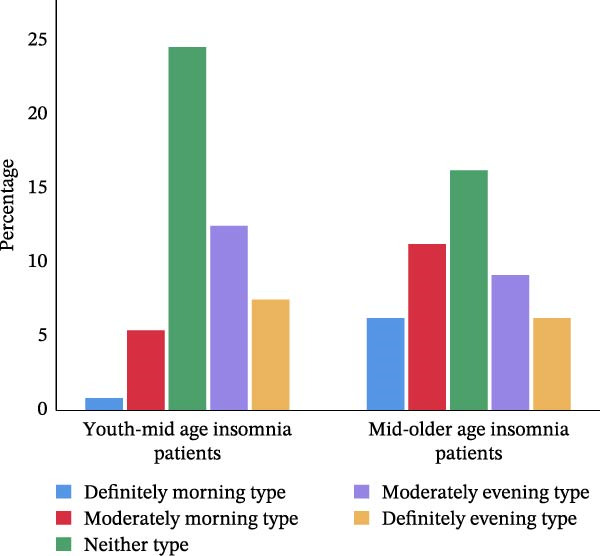
Differences in morningness–eveningness chronotype between young adult age insomnia patients and old adult age patients. Evening types were more prevalent in the young adult group (young adult group: 39.4%; old adult group: 31.3%), while morning types were more prevalent in the old adult group (young adult group: 12.3%; old adult group: 23.8%).

In terms of questionnaire scores, two‐sample Student’s *t*‐tests revealed significant differences between the two groups of insomnia patients in MEQ scores (young adult group: 44.3 ± 13.1; old adult group: 50.5 ± 15.7) and STAI scores (young adult group: 86.1 ± 22.5; old adult group: 79.5 ± 20.6), while no group differences were found in the scores of other scales. Further analysis showed that in STAI scores, state anxiety scores were higher in the young adult group compared to the old adult group (young adult group: 41.6 ± 12.2; old adult group: 37.4 ± 10.9), while trait anxiety scores showed no statistical difference between the two groups. After converting MEQ scores into categorical variables (Figure 1), a chi‐square test found that evening types were more prevalent in the young adult group (young adult group: 39.4%; old adult group: 31.3%), while morning types were more prevalent in the old adult group (young adult group: 12.3%; old adult group: 23.8%).

In terms of HRV parameters, significant differences were observed in all HRV parameters between the two groups of insomnia patients, except for HF, according to the results of two‐sample Student’s *t*‐tests. More information on the differences between the two groups in questionnaire scores and HRV indices can be found in Table 1.

**Table 1:** Differences in questionnaire scores and heart rate variability parameters between young and old adult insomnia patients.

Variables	Young adult insomnia patients	Old adult insomnia patients	*p* value
AIS	11.5 ± 3.7	12.4 ± 4.4	0.088
ISI	16.9 ± 5	16.9 ± 5.3	0.995
ESS	6.3 ± 4.3	6.4 ± 4.4	0.843
HAMA	10.5 ± 6.2	10.3 ± 7.4	0.761
HAMD	12.7 ± 5.9	13.2 ± 7.1	0.53
FS‐14	8.9 ± 4.8	8.2 ± 4.6	0.274
Physical fatigue	5.5 ± 2.3	5.2 ± 2.8	0.311
Psychological fatigue	3.4 ± 3.9	3 ± 2.8	0.447
MEQ	44.3 ± 13.1	50.5 ± 15.7	**0.001** ^∗^
STAI	86.1 ± 22.5	79.5 ± 20.6	**0.016** ^∗^
State anxiety	41.6 ± 12.3	37.4 ± 10.9	**0.005** ^∗^
Trait anxiety	44.5 ± 11.5	42 ± 10.8	0.084
TCSQ	68.6 ± 14.5	72.6 ± 14.8	0.053
Positive coping style	31.8 ± 9.9	33.9 ± 8.8	0.09
Negative coping style	36.8 ± 11.7	38.7 ± 13.4	0.231
SDNN (ms)	40.4 ± 18.9	28.8 ± 14.9	**<0.001** ^∗^
SDSD (ms)	23.9 ± 16.6	19.1 ± 13.9	**0.015** ^∗^
RMSSD (ms)	24 ± 16.6	19.2 ± 14.1	**0.015** ^∗^
NN50 (ms)	6.3 ± 9.6	2.9 ± 4.9	**0.001** ^∗^
pNN50 (ms)	2.5 ± 3.8	1.1 ± 1.9	**0.001** ^∗^
RRIs (ms)	751.5 ± 116.4	800.8 ± 110.3	**0.001** ^∗^
TP (ms^2^)	1057.2 ± 1132.2	510.2 ± 611.2	**<0.001** ^∗^
VLF (ms^2^)	496.1 ± 196.4	196.4 ± 185.8	**<0.001** ^∗^
HF (ms^2^)	169.6 ± 361.9	116.4 ± 205.1	0.161
LF (ms^2^)	387.2 ± 431	197.4 ± 321.6	**<0.001** ^∗^
LF/HF	5.5 ± 5.7	3.7 ± 3.8	**0.005** ^∗^

*Note:* HF, high‐frequency power; LF, low‐frequency power; NN50, number of consecutive R–R intervals differing by >50 ms; pNN50, the percentage value of NN50 intervals; RMSSD, the root mean square of successive RRI differences; SDNN, the standard deviation of RRIs; SDSD, the root mean square of RRIs; VLF, very‐low‐frequency power. Bold numbers indicate significant differences between groups. The “ ^∗^” means significant group differences in these variables.

Abbreviations: AIS, Athens Insomnia Scale; ESS, Epworth Sleepiness Scale; FS‐14, Fatigue Scale‐14; HAMA, Hamilton Anxiety Rating Scale; HAMD, Hamilton Depression Rating Scale; ISI, Insomnia Severity Index; MEQ, Morningness–Eveningness Questionnaire; RRIs, R–R intervals; STAI, State–Trait Anxiety Inventory; TCSQ, Trait Coping Style Questionnaire; TP, total power.

### 3.3. Predictors of Insomnia Severity With Questionnaire Scores and HRV Parameters

In the young adult group, LASSO regression followed by forward stepwise linear regression refined two significant predictors of the AIS score (Table 2). The linear regression model demonstrated moderate fit with an adjusted *R* ^2^ of 0.161 (*F* = 12.84, *p* < 0.001). Whereas LASSO regression analysis followed by forward stepwise linear regression refined three significant predictors of the ISI score (Table 2). The linear regression model demonstrated moderate fit with an adjusted *R* ^2^ of 0.2 (*F* = 11.23, *p* < 0.001).

**Table 2:** Variables for predicting insomnia severity with questionnaire scores and heart rate variability parameters by linear regression.

Dependent variables	Independent variables	*β* values	95% CI lower limit	95% CI upper limit	*p* value
Young adult insomnia patients’ group					
AIS	HAMD score	0.389	0.224	0.55	<0.001
AIS	MEQ score	0.184	0.021	0.356	0.028
ISI	FS‐14 score	0.43	0.228	0.509	<0.001
ISI	HAMD score	0.232	0.077	0.423	0.005
ISI	MEQ score	0.189	0.028	0.389	0.024
Old adult insomnia patients’ group					
AIS	HAMD score	0.334	0.111	0.553	0.004
AIS	HAMA score	0.274	0.051	0.492	0.016
AIS	LF/HF ratio	−0.198	−0.481	−0.073	0.008
ISI	HAMA score	0.55	0.377	0.656	<0.001
ISI	RRIs	−0.168	−0.341	−0.021	0.027

*Note:* HF, high‐frequency power; LF, low‐frequency power.

Abbreviations: AIS, Athens Insomnia Scale; CI, confidence interval; FS‐14, Fatigue Scale‐14; HAMA, Hamilton Anxiety Rating Scale; HAMD, Hamilton Depression Rating Scale; ISI, Insomnia Severity Index; MEQ, Morningness–Eveningness Questionnaire; RRIs, R–R intervals.

In the old adult group, LASSO regression followed by forward stepwise linear regression refined three significant predictors of the AIS score (Table 2). The linear regression model demonstrated moderate fit with an adjusted *R* ^2^ of 0.352 (*F* = 22.54, *p* < 0.001). While LASSO regression followed by forward stepwise linear regression refined two significant predictors of the ISI score (Table 2). The linear regression model demonstrated moderate fit with an adjusted *R* ^2^ of 0.333 (*F* = 30.71, *p* < 0.001).

Post hoc power analysis was performed using 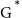
Power 3.1 to verify the adequacy of sample size for the primary regression models. For the young adult group (*n* = 124) and old adult group (*n* = 120), we input *α* = 0.05 (two‐tailed), effect size *f* ^2^ = 0.15 (medium effect, derived from adjusted *R* ^2^ = 0.161–0.352), and the number of predictors (*k* = 2–3 per model). The analysis indicated that the study had 95%–98% power to detect the observed associations between predictors (e.g., HAMD, MEQ, HAMA, and LF/HF ratio) and insomnia severity (AIS/ISI scores), which is sufficient for observational studies exploring predictor‐outcome relationships.

## 4. Discussion

The present study investigated age‐related differences in clinical characteristics, autonomic nervous system function, and factors associated with insomnia severity in primary insomnia patients. By dividing participants into young adult (18–44 years) and old adult (45+ years) groups, we identified distinct patterns in anxiety symptoms, chronotype, and HRV parameters, as well as divergent predictors of insomnia severity between the two groups.

### 4.1. Age‐Related Differences in Anxiety and Chronotype

Young adult‐age patients exhibited significantly higher state anxiety scores (STAI‐State) than old adult‐age patients, suggesting that transient, situation‐specific anxiety may be more prominent in younger adults with insomnia. This finding aligns with prior research [12], indicating that younger individuals often face higher stress levels from work, family, or social responsibilities, which can exacerbate state anxiety and disrupt sleep regulation. Notably, trait anxiety did not differ between groups, implying that dispositional anxiety may be a stable trait across age groups, while situational factors play a more dynamic role in younger patients.

Regarding chronotype, the young adult group had a higher prevalence of evening types, whereas old adult‐age patients were more likely to be morning types. This aligns with the natural shift toward morningness with aging, potentially driven by biological clock adjustments and reduced evening alertness [34–36]. Evening chronotype in younger adults may contribute to insomnia through delayed sleep–wake cycles, social jet lag, or irregular sleep schedules, which are known risk factors for poor sleep quality [37].

### 4.2. Autonomic Nervous System Dysfunction in Insomnia

HRV analysis revealed significant age‐related differences in autonomic functions. Young adult‐age patients showed higher SDNN, RMSSD, and LF power (except HF), indicating increased overall HRV and sympathetic‐parasympathetic activity. Elevated HRV in younger insomnia patients may reflect heightened physiological arousal or stress responsiveness, consistent with their higher state anxiety scores [38]. In contrast, old adult age patients exhibited a stronger association between the LF/HF ratio (a marker of sympathetic‐vagal balance) and insomnia severity (AIS/ISI scores), suggesting that age‐related declines in autonomic flexibility or cardiovascular regulation may contribute to sleep disturbances in this group [8]. Reduced sympathetic‐vagal balance (lower LF/HF ratio) in older adults has been linked to chronic inflammation and cardiovascular comorbidities, which may interact with insomnia to worsen outcomes [39].

### 4.3. Predictors of Insomnia Severity: Age‐Specific Mechanisms

In young adult patients, depression (HAMD), psychological fatigue (FS‐14), and MEQ scores were significant predictors of AIS/ISI severity. This highlights the role of psychological distress (e.g., depressive symptoms) and chronotype misalignment in younger individuals. Depressive symptoms often coexist with insomnia in a bidirectional relationship, as demonstrated by numerous studies [40–43], while psychological fatigue may reflect poor sleep restorativeness or lifestyle factors like sedentary behavior [44]. The association with MEQ scores further underscores the importance of circadian rhythm regulation in younger patients, suggesting that interventions targeting sleep timing (e.g., light therapy and chronotherapy) may be particularly beneficial [45, 46].

For old adult patients, anxiety (HAMA), depression (HAMD), and HRV parameters (LF/HF ratio and RRIs) emerged as key predictors. The stronger link between anxiety/trait depression and insomnia in this group may reflect cumulative lifetime stressors or age‐related neurochemical changes (e.g., reduced serotoninergic function) [47]. The negative association between RRIs (mean heart rate) and ISI scores is intriguing; slower heart rate in older adults might indicate compromised autonomic regulation, consistent with findings with the LF/HF ratio [48]. These results suggest that autonomic dysfunction plays a more mechanistic role in older adults’ insomnia, potentially mediated by cardiovascular or neurodegenerative processes.

### 4.4. Clinical Implications and Limitations

The divergent predictors of insomnia severity between age groups have important clinical implications. Younger patients may benefit from interventions targeting anxiety, depression, and circadian alignment (e.g., cognitive–behavioral therapy for insomnia [CBT‐I], light therapy), while older adults may require approaches addressing autonomic regulation (e.g., stress management, cardiovascular risk reduction) alongside psychological interventions. Notably, interventions targeting autonomic regulation have shown promise for older adults with insomnia; for example, high‐resolution, relational, resonance‐based, electroencephalic mirroring (HIRREM) has been shown to reduce insomnia symptoms while improving HRV and autonomic balance [49]. Combined with psychological interventions (e.g., CBT‐I), such approaches may address the dual burden of anxiety and autonomic dysfunction in older patients, aligning with our findings that these factors are key predictors of insomnia severity. However, a major limitation of this cross‐sectional study is the inability to determine the direction of causality between insomnia and abnormal HRV. Chronic insomnia may induce autonomic dysregulation via sustained stress responses, while preexisting autonomic dysfunction could also predispose individuals to insomnia; longitudinal studies tracking both variables over time are needed to disentangle this dilemma. The exclusion of comorbid conditions (e.g., mild hypertension) in old adults with insomnia may affect generalizability. Future longitudinal studies should include insomnia patients with comorbidities, incorporating stratified analyses or adjusting for comorbidity burden to validate our results in more representative populations, which are needed to validate these associations and explore age‐specific therapeutic pathways. Another potential limitation of this study is the overrepresentation of female participants, which may limit the generalizability of our findings to male populations with primary insomnia. Gender differences in sleep physiology, psychological stress responses, and autonomic function are well‐documented, and future studies should aim for balanced gender recruitment to explore whether the age‐specific predictors identified herein vary by gender. Notably, some HRV differences observed may reflect age‐related physiological changes independent of insomnia; future studies could include a healthy control group to disentangle the unique effects of insomnia from normal aging on autonomic function. And a 10‐min recording for HRV is necessary for future studies. We did not collect data on prior trauma (physical or nonphysical) or PTSD symptoms, which may confound the associations between age, HRV, and insomnia severity. Emerging evidence highlights the role of trauma in shaping brain‐autonomic nervous system interactions relevant to sleep [50], and future studies should incorporate trauma assessments (e.g., PTSD) to clarify whether trauma mediates or modifies the age‐specific relationships identified herein. For younger adults, jet lag may mediate the association between evening chronotype and insomnia, as noted in our findings; future studies could measure social jet lag explicitly to test this pathway. Additionally, trauma‐related autonomic alterations may contribute to both insomnia and HRV abnormalities in both age groups, highlighting the need for multidimensional assessments in future research. Stratifying patients by the severity of insomnia, rather than looking for age‐related changes, is another valuable direction in the future. Based on the present study, we stratified patients by insomnia severity within each age group and found lower VLF and LF values in patients with moderate/severe insomnia than those with mild insomnia within the old age group (Table S1), and further detailed research requires a larger sample size. The lack of a priori power analysis prior to study initiation. Although post hoc analysis confirmed sufficient power to detect medium‐to‐large effects, future studies should conduct a priori calculations to optimize the sample size and minimize the risk of type II errors.

In conclusion, this study highlights age‐dependent mechanisms underlying primary insomnia, with younger patients influenced by psychological and circadian factors and older patients by autonomic dysfunction and chronic anxiety/depression. These findings underscore the need for age‐tailored approaches in diagnosing and treating insomnia, emphasizing the integration of psychological, behavioral, and physiological interventions.

## Author Contributions

Kong Xinyu and Guo Xiaodan collect the data and write the original manuscript. Wang Mengmeng and Zhang Haodong analyze the data. Xiao Fulong designs the study and reviews the manuscript.

## Funding

This work was supported by the National Natural Science Foundation of China (Grant 8247010651).

## Disclosure

The authors have nothing to report.

## Conflicts of Interest

The authors declare no conflicts of interest.

## Supporting Information

Additional supporting information can be found online in the Supporting Information section.

## Supporting information


**Supporting Information** Table S1: The differences in heart rate variability parameters between mild and moderate/severe insomnia patients in young and old adult insomnia patients.

## Data Availability

Anonymized data not published within this article will be available from the corresponding author upon reasonable request.
