# Supplementary material for: Age Differences in Factors Associated With Insomnia Severity: A Comparative Study of Questionnaire Scores and Heart Rate Variability Parameters
Source: Depress Anxiety. 2026 Jul 3;2026:7711037. doi: 10.1155/da/7711037 (PMC13329830; doi:10.1155/da/7711037)
Supplement: Supplementary file 1 — Supporting Information Table S1: The differences in heart rate variability parameters between mild and moderate/severe insomnia patients in young and old adult insomnia patients. [file DA-2026-7711037-s001.docx]

**Supplementary Table 1 Differences in heart rate variability parameters between mild and moderate/severe insomnia patients in young and old adult insomnia patients**

| **Variables** | **Mild insomnia patients** | **Moderate/severe insomnia patients** | **P value** |
| --- | --- | --- | --- |
| **Young adult insomnia patients** | | | |
| **Insomnia stratified by AIS score** | | | |
| **SDNN (ms)** | 43±22.9 | 38.9±16.1 | 0.498 |
| **SDSD (ms)** | 26.7±20.3 | 22.2±13.8 | 0.561 |
| **RMSSD (ms)** | 26.7±20.3 | 22.4±13.7 | 0.634 |
| **NN50 (ms)** | 8.4±11.8 | 5.1±7.7 | 0.431 |
| **pNN50 (ms)** | 3.3±4.6 | 2±3.1 | 0.431 |
| **RRIs (ms)** | 750.7±82.6 | 751.9±132.9 | 0.776 |
| **TP (ms^2^)** | 1298.8±1593.5 | 914.7±712.8 | 0.488 |
| **VLF (ms^2^)** | 576.5±807.5 | 448.7±380 | 0.653 |
| **HF (ms^2^)** | 236.2±487.1 | 130.4±257.7 | 0.733 |
| **LF (ms^2^)** | 474.6±574.5 | 335.6±310.8 | 0.559 |
| **LF/HF** | 5.8±7.5 | 5.3±4.4 | 0.924 |
| **Insomnia stratified by ISI score** | | | |
| **SDNN (ms)** | 42.7±22.1 | 39.3±17.2 | 0.401 |
| **SDSD (ms)** | 25.6±18 | 23.1±15.9 | 0.612 |
| **RMSSD (ms)** | 25.6±18 | 23.3±15.9 | 0.682 |
| **NN50 (ms)** | 7±10.6 | 5.9±9.1 | 0.861 |
| **pNN50 (ms)** | 2.8±4.2 | 2.3±3.6 | 0.861 |
| **RRIs (ms)** | 748.5±82.6 | 752.9±129.9 | 0.677 |
| **TP (ms^2^)** | 1249.7±1537.5 | 914.7±712.8 | 0.41 |
| **VLF (ms^2^)** | 582.9±776.1 | 454.8±452.7 | 0.348 |
| **HF (ms^2^)** | 197.5±345.4 | 156.4±370.9 | 0.65 |
| **LF (ms^2^)** | 456.1±597.2 | 354.3±322.9 | 0.769 |
| **LF/HF** | 5.9±8.5 | 5.2±3.8 | 0.325 |
| **Old adult insomnia patients** | | | |
| **Insomnia stratified by AIS score** | | | |
| **SDNN (ms)** | 20.7±14.5 | 27.2±14.3 | 0.082 |
| **SDSD (ms)** | 22.2±13.8 | 18.2±13.7 | 0.157 |
| **RMSSD (ms)** | 21.1±14.7 | 18.2±13.7 | 0.128 |
| **NN50 (ms)** | 3.1±4.4 | 2.8±5.1 | 0.483 |
| **pNN50 (ms)** | 1.2±1.7 | 1.1±2 | 0.483 |
| **RRIs (ms)** | 810.1±126 | 796.2±102.1 | 0.563 |
| **TP (ms^2^)** | 608.4±745.3 | 461.1±530.3 | 0.065 |
| **VLF (ms^2^)** | 230.9±184.9 | 179.1±185 | **0.019*** |
| **HF (ms^2^)** | 122.9±227.7 | 113.2±194.2 | 0.324 |
| **LF (ms^2^)** | 254.1±390.6 | 169.1±279.3 | **0.026*** |
| **LF/HF** | 4.9±5.6 | 3.1±2.4 | 0.199 |
| **Insomnia stratified by ISI score** | | | |
| **SDNN (ms)** | 28.9±10.9 | 28.7±16.6 | 0.374 |
| **SDSD (ms)** | 18.9±20.4 | 19.1±15.5 | 0.339 |
| **RMSSD (ms)** | 19±10.5 | 19.3±15.5 | 0.351 |
| **NN50 (ms)** | 3.3±5.3 | 2.7±4.6 | 0.661 |
| **pNN50 (ms)** | 1.3±2.1 | 1.1±1.8 | 0.661 |
| **RRIs (ms)** | 819.3±117.8 | 791.9±106.2 | 0.288 |
| **TP (ms^2^)** | 460.9±340.4 | 533.9±706.4 | 0.454 |
| **VLF (ms^2^)** | 201.9±166.3 | 193.8±195.5 | 0.288 |
| **HF (ms^2^)** | 90.3±113.8 | 129±236.4 | 0.64 |
| **LF (ms^2^)** | 165±151 | 213±377.2 | 0.646 |
| **LF/HF** | 3.6±3.2 | 3.7±4.1 | 0.867 |

Abbreviation: AIS, Athens Insomnia Scale; ISI, Insomnia Severity Index; RRIs, R-R intervals; SDNN, the standard deviation of RRIs; SDSD, the root mean square of RRIs; RMSSD, the root mean square of successive RRI differences; NN50, number of consecutive R-R intervals differing by > 50 msec; pNN50, the percentage value of NN50 intervals; TP, total power; HF, high frequency power; LF, low frequency power; VLF, very low frequency power.
